# Assessing Fish Immunotoxicity by Means of *In Vitro* Assays: Are We There Yet?

**DOI:** 10.3389/fimmu.2022.835767

**Published:** 2022-02-28

**Authors:** Helmut Segner, Kristina Rehberger, Christyn Bailey, Jun Bo

**Affiliations:** ^1^Centre for Fish and Wildlife Health, Department of Pathobiology and Infectious Diseases, Vetsuisse Faculty, University of Bern, Bern, Switzerland; ^2^Fish Immunology Group, CISA-INIA, Madrid, Spain; ^3^Laboratory of Marine Biology and Ecology, Third Institute of Oceanography, Xiamen, China

**Keywords:** fish, immunotoxicity, *in vitro*, toxicity screening, ecotoxicological hazard assessment, comparative immunity, fish immune cells

## Abstract

There is growing awareness that a range of environmental chemicals target the immune system of fish and may compromise the resistance towards infectious pathogens. Existing concepts to assess chemical hazards to fish, however, do not consider immunotoxicity. Over recent years, the application of *in vitro* assays for ecotoxicological hazard assessment has gained momentum, what leads to the question whether *in vitro* assays using piscine immune cells might be suitable to evaluate immunotoxic potentials of environmental chemicals to fish. *In vitro* systems using primary immune cells or immune cells lines have been established from a wide array of fish species and basically from all immune tissues, and in principal these assays should be able to detect chemical impacts on diverse immune functions. In fact, *in vitro* assays were found to be a valuable tool in investigating the mechanisms and modes of action through which environmental agents interfere with immune cell functions. However, at the current state of knowledge the usefulness of these assays for immunotoxicity screening in the context of chemical hazard assessment appears questionable. This is mainly due to a lack of assay standardization, and an insufficient knowledge of assay performance with respect to false positive or false negative signals for the different toxicant groups and different immune functions. Also the predictivity of the *in vitro* immunotoxicity assays for the *in vivo* immunotoxic response of fishes is uncertain. In conclusion, the currently available database is too limited to support the routine application of piscine *in vitro* assays as screening tool for assessing immunotoxic potentials of environmental chemicals to fish.

## Immunotoxicology Has Relevance for the Health of Fish Populations

In assessing the risk of environmental contaminants for fish, for a long time most consideration has been given to apical effects such as changes of survival, growth and reproduction (1, 2). This approach is based on the assumption that the apical effects are predictive for changes in population growth, although this assumption is partly questionable [e.g., (1, 3–5)]. More recently, a paradigm shift has taken place giving more emphasis on the role of sublethal effects for the ecological impact of toxicants [e.g., (2, 6–11)]. Particularly chemicals with specific modes of actions, for instance, pharmaceuticals, are unlikely to cause apical effects at environmental concentrations, still these concentrations may be high enough to modify physiological performance and life history traits of exposed organisms, what can have consequences for organism fitness and population growth.

To make sublethal effects assessment a valuable addition in evaluating the risks of environmental contaminants to fish populations, it is critical to not get lost in measuring an increasingly broad range of subtle parameters, but to focus on processes and traits that have potential toxicological and ecological inferences, and/or influence the vulnerability of species and populations towards chemical impacts (2, 8, 12, 13). In the present communication, we focus on the assessment of immunological effects of chemicals in fish. Immunity is directly linked to phenotypic fitness, survival probability and evolutionary selection (14–16). An immune system that is able to execute its basic functions, i.e. recognition and response, is essential for maintaining the integrity of the organism, and it prevents damage as it may arise from infectious pathogens or other environmental stressors including toxic chemicals (17). Environmental contaminants can interfere with the immune system of exposed organisms and this may result in immune dysfunction. Chemical effects on the immune system are usually not immediately lethal, although prolonged disturbances of immune homeostasis such as, e.g., chronic inflammation are known to be associated with increased morbidity and mortality (18–20). In addition, immune dysfunction is both a predisposing and enabling factor for pathogen-induced diseases and mortalities (4). Finally, since immune responses are costly, they may trade-off with other fitness-relevant life history traits (21), and through this mechanism, immune disturbances can indirectly impair reproduction and growth, reduce overwinter survival, or cause debilitation thereby increasing the risk of predation (12, 22–25).

Fish immunotoxicology aims to understand the impact of environmental contaminants on the health of fish and to assess the consequences for fish populations (26–30). There exists broad evidence that immunoactive chemicals are a relevant thread to fish populations. A wide array of environmental contaminants including legacy compounds, endocrine disrupting compounds (EDCs), pesticides, metals, and pharmaceuticals have been shown to impact the immune system of fishes [for reviews cf. (31–33)]. The International Council for the Exploration of the Sea (ICES) concluded that almost all known chemicals seem to impact the immune system of fishes (34). Also nanoparticles are known to interfere with fish immunity [for review see (35)]. Immune disturbances have been observed worldwide in fish populations living in contaminated habitats [e.g., (36–40)]. With respect to effluents from wastewater treatment works, which are major point sources of aquatic contamination, a number of studies reported immunotoxic effects for fish [e.g., (41, 42)]. Liney et al. (43) observed that the immunotoxic effects of wastewater effluents occurred at concentrations lower than those required to induce recognizable changes in the structure and function of the reproductive endocrine system. Similarly, Rehberger et al. (44) as well as Kernen et al. (45) reported that low concentrations of ethinylestradiol, a major environmental EDC, which disrupt reproductive functions of exposed fish, did also disrupt the immune functions. Collectively, the available findings indicate that chemically induced immunotoxicity is not an artifact of high dose laboratory experiments but that it is a relevant environmental hazard. Importantly, chemical effects on the fish immune system may cumulate with the impacts of other environmental stressors targeting the immune system, for instance, increasing water temperature or stress caused by habitat degradation (46, 47).

Given that immunotoxicity is a relevant ecotoxicological issue, the question is how to detect immunotoxic activities of environmental contaminants to fish? A range of methods and assays to investigate chemical impacts on the fish immune system is available [e.g., (26, 48, 49)], but currently there exist no generally accepted and/or standardized test procedures for assessing potential immunotoxic activities of chemicals to fish. Immunotoxicity appears to be a kind of “blind spot” in ecotoxicological hazard assessment. Hazard profiling of chemicals and other environmental agents relies on a diversity of methods, including computational techniques such as read-across or (Quantitative) Structure Activity Relationships (QSAR), on comparative approaches, i.e. using information from mammalian hazard testing for non-mammalian species, but also *in vitro* assays can play an important role. In fact, over recent years, *in vitro* assays have been increasingly applied for screening purposes in (eco)toxicological hazard assessment [e.g. (50–53)]. Immunotoxicity, however, was not or only marginally considered in these approaches, although a group of *in vitro* assays – in the sense of “Invitroomics” (54) - may well be able to test for the various immune targets potentially affected by toxic agents. The question to be addressed in this context is whether fish cell-based *in vitro* assays are indeed suitable and sufficient to evaluate the immunotoxicity of environmental chemicals. The aim of the present communication is to critically review the current state of knowledge on *in vitro* fish immunotoxicity testing. To this end, we will discuss what kind of *in vitro* assays are available using fish immune cells, for what toxicological purposes they have been applied, and what their strengths and weaknesses are.

## Assessing the Immunotoxic Activity of Chemicals by *In Vitro* Approaches

Chemical impacts on immunity include both immunosuppression and immunostimulation (55, 56). The former can result in increased susceptibility to pathogen infections and pathogen-induced mortalities, activation of opportunistic microorganisms, or development of neoplasia (56); the latter can result in hypersensitivity reactions and autoimmune disorders – a response that has been frequently observed in man, but not yet reported for fish. Chemical-induced immunomodulation can have also indirect effects, for instance *via* resource trade-offs or endocrine-immune interactions, on other life history traits such as growth, reproduction and behaviour (57–59). Importantly, not each chemical-induced disturbance of an immune parameter will result in impaired immune functioning and competence. Only if the induced immune modulation is of sufficient strength and quality, an adverse effect will result (55, 60).

The immune system is, like the endocrine system, a highly complex system composed of a huge diversity of organs, cells, and mediator as well as effector molecules. The diffuse organization and the integrated functioning of the immune system complicates immunotoxicity assessment as it raises questions which targets within the system are impacted by the action of a chemical. A further complicating fact in immunotoxicity assessment is that the overall capacity of the immune system to maintain homeostasis and health as well as to defend the organism against external stressors depends not so much on an individual immune element but on the balance between individual components and the functioning at the systemic level. By means of networked self-control the immune system is able to buffer to some extent stressor-induced disturbances, and this makes it difficult to define thresholds of adversity or to extrapolate from the toxicant-induced modulation of a specific immune component to an overall impairment of immune functionality and capacity (60). In addition, toxic impacts often may not be visible in the resting immune system, but only when the immune system is activated, e.g., after a pathogen challenge (60, 61). Finally, immune responses may not be directly triggered by a toxicant, but as indirect response to other forms of toxicity. For instance, an inflammatory reaction may be a secondary response to a toxicant-induced tissue damage (62). Also toxicant effects on the microbiome can have consequences for immune system function [e.g., (63)].

From what has been said above, it is evident that *in vitro* assays will be able to test only for certain forms of chemical impacts on the immune system (60, 64). *In vitro* assays cannot take into account for indirect effects such as neuro-immune-endocrine interactions, and they cannot reflect integrated responses resulting from the interactions of the individual immune components. However, *in vitro* assays can detect direct effects of chemicals on immune cell viability or proliferation. They can also distinguish if the chemical actions are selective for specific immune cell types. In addition, *in vitro* assays can test for the interference of chemicals with immune cell functioning, for instance, changes in signalling pathways, in the transcriptome, in oxidative burst or phagocytic activity, in antibody production, or in the production and release of soluble mediators such as cytokines. A challenge for *in vitro* immunotoxicity assessment, however, remains the fact that the immune system is composed of a diversity of cell types. Innate immunity involves, among others, granulocytes, neutrophils, macrophages, natural killer cells, or mast cells, while adaptive immunity involves B and T cells. In addition, macrophages and dendritic cells which act as antigen-presenting cells are linking the innate and adaptive arms of the immune system. Chemicals may target any of these immune cell types and their functions, and *in vitro* tests methodologies must be designed in a way to be able to assess the potential diversity in targets (65).

The use of *in vitro* assays for immunotoxicity testing is most advanced in human toxicology. Currently, immunotoxicity testing in human toxicology relies on animal tests, which include general immune endpoints in repeated dose studies and trigger-based tests on a case-by-case basis [e.g., (66–68)]. In the United States, the National Toxicology Program developed a tiered *in vivo* immunotoxicity testing strategy which includes tier I tests like the assessment of antigen-induced humoral immunity or cell-mediated immunity as well as general parameters like immune organ weight or histopathology (69). Tier II includes, among others, tests on hypersensitivity or on the cytotoxic T cell response. A tiered strategy for *in vitro* immunotoxicity assessment could start with an evaluation of myelotoxicity, which examines whether the toxicant leads to a decreased production of bone marrow-derived immune progenitor cells (64, 68, 70). For this purpose, often the humane/murine clonogenic CFU-GM (Colony Forming Unit – Granulocyte Macrophage) test is used (71). If a compound inhibits the proliferation of progenitor cells, it can be considered to be immunotoxic and a further evaluation is not needed. If a compound is not myelotoxic, the following tiers assess the impact of the chemical on the viability and function of differentiated immune cells, mainly on lymphocytes. Initially, the overt cytotoxicity of the chemical towards immune cells is determined, and then, using non-cytotoxic concentrations, the impacts on selected cell functions are tested, for instance, cytokine production or lymphocyte proliferation assays (64, 68, 70). The function tests include also genetically modified reporter gene cell systems like the “fluorescent cell chip” (72) or the IL-2 luciferase assay (73). Despite the fact that meanwhile a diversity of cell-based *in vitro* immunotoxicity assays is available and characterized, they are usually not yet included in the regulatory testing schemes of human toxicological risk assessment (60, 68).

Most success of *in vitro* immunotoxicity assays has been achieved in the area of chemical-induced immunostimulation such as skin sensitization. Here, *in vitro* assays are integrated in the assessment approach in the context of Adverse Outcome Pathways (AOP). The AOP framework intends to quantitatively link molecular initiating events of toxicity through a series of “key events” to adverse outcomes (74, 75). The AOP for skin sensitization starts with covalent interactions of the irritant with skin proteins as molecular initiating event, and then proceeds through a series of immune-related key events to end up in inflammation and allergic dermatitis. The immune key events which mechanistically link the MIE to the adverse outcome are induction of inflammatory cytokines, activation of dendritic cells, and activation and proliferation of T cells. For these immune responses, *in vitro* assays are available that provide quantitative concentration-response data to be integrated into the risk assessment of the skin sensitizing activity of chemicals (76).

One aspect that must not be neglected when using *in vitro* assays for immunotoxicity testing are technical issues. For instance, physicochemical characteristics of the test material or vehicle solvent may interfere with the *in vitro* systems. Also the influence of serum, as often used in cell cultures, on the bioavailability of the test chemicals, can be a confounding factor.

Finally, it is important to distinguish between *in vitro* and *ex vivo* assays. In the latter case, animals are exposed *in vivo* to the suspected immunotoxicant, and after the *in vivo* treatment, immune cells are isolated and tested for their functioning. Although the measurements on the isolated cells are done *in vitro*, the experiment still represents an *in vivo* study, because conditioning and treatment of the immune cells was done in the intact animal. The present communication will deal only with assays that do not involve treatments of animals, i.e. *ex vivo* assays are not considered.

## Fish Immune Cells *In Vitro*: Cell Lines and Primary Cells

*In vitro* systems that have been used to study toxicant effects on fish immunity include mainly cell lines and primary cells, either in fresh suspensions or in primary culture. Therefore we will focus on these systems. Cells isolated from organs or tissues of an organism are primary cells; they typically are maintained for a few hours - usually as suspension – or for a few days – then either as two-dimensional monolayer culture or three-dimensional aggregate culture (77, 78). By convention, the primary cell system ends and a cell line arises at the time of the first subculture (54, 79). The cell lines proliferate *in vitro* and after a certain time period they are split up into subcultures, a process often referred to as passaging (78). Finite cell lines undergo only a limited number of passaging into subcultures, whereas continuous cell lines grow indefinitely (80, 81). In addition to primary cells and cell lines, also other *in vitro* systems like tissue explants could be used for immunotoxicity studies, but to date this interesting methodology has been rarely applied to immune organs (82).

### Piscine Immune Cell Lines

First introduced in the 1960s (83), the number of fish cell lines is continuously growing since then. While Fryer and Lannan (84) listed 152 fish cell lines, Lakra et al. (85) identified 283 cell lines, and these numbers keep increasing. Fish cell lines have been frequently used for the diagnosis of fish diseases as well as to study immunological responses [e.g., (54, 86, 87)]. **Table 1** lists examples of cell lines derived from fish immune tissues including cell lines for which immunological functions have been demonstrated and cell lines for which no such characterization is available. The fact that a cell line originates from immune organs does not necessarily mean that it expresses immune functions. On the other hand, cell lines derived from non-immune tissues may display immune features such as lipopolysaccharide (LPS)- or cortisol-inducible immune gene expression (113, 114). Still, the use of cell lines derived from immune tissues and characterized for their immunological profile, like e.g. the macrophage line RTS11 (105, 106) appears to be preferable for immunological research and testing.

**Table 1:** Examples of fish cell lines derived from immune tissues.

Species	Tissue of origin	Cell line designation	Morphology	Immune-specific markers and functions	Reference
*Acipenser transmontanus*	Spleen	WSS-2		Not assessed	(88)
*Acipenser baerii*	Head kidney		Polynucleated, polygonal	Not assessed	(89)
*Anguilla anguilla*	Trunk kidney	EK	Fibroblast-like	Constitutive and poly I:C inducible expression of immune-related genes	(90)
*Anguilla rostrata*	Peripheral blood leukocytes	PBLE	Fibroblast-like	No respiratory burst activity; probably arising from mesenchymal stem cell	(91)
*Carassius auratus*	Trunk kidney	GMLC	Macrophage-like	Production of nitric oxide. Phagocytic and respiratory burst activity; responsive to lipopolysaccharide (LPS)	(92)
*Catla catla*	Thymus	CTM	Macrophage-like	Production of lysozyme and nitric oxide. Phagocytic and repiratory burst activity. Expression of Fc receptor	(93)
*Cyprinus carpio*	Peripheral blood	CLC	Macrophage-like	Respiratory burst activity	(94, 95)
*Ictalurus puncatus*	Peripheral blood	1B10	Lymphoblast-like	B cell-like properties; production of cytoplasmic and membrane IgM	(96)
*Ictalurus punctatus*	Peripheral blood	Several clonal cell lines	Cytotoxic- and NK cell-like	Cytotoxic activities, partly TCRαβ-positive, presence of putative Fc receptor for IgM	(97, 98)
*Ictalurus punctatus*	Peripheral blood	C24, K2, M22, Z33	Monocyte-like	LPS-inducible IL-1 production, phagocytic activity, antigen presenting function	(99)
*Epinephelus akaara*	Spleen	EAGS	Fibroblast-like	Not assessed	(100)
*Oncorhynchus mykiss*	Spleen (explants)		Phagocyte-like	Phagocytic activity, phagocyte-like cytochemical staining properties	(101)
*Oncorhynchus mykiss*	Trunk kidney	RTK	Fibroblast-like	Not assessed	(102)
*Oncorhynchus mykiss*	Head kidney	TPS	Fibroblastic, epitheloid	stromal cell line with no immune capacity but supporting hematopoiesis in immune cell populations	(103)
*Oncorhynchus mykiss*	Spleen		Epitheloid, fibroblastic	Phagocytic activity in about 20% of the cells	(104)
*Oncorhynchus mykiss*	Spleen	RTS11	Macrophage-like	Phagocytic activity, responsive to LPS; LPS- inducible expression of macrophage-typical genes	(105, 106)
*Oreochromis mossambicus*	Head kidney	THK	Fibroblastoid	Expression of monocyte/macrophage-type transcripts; properties of melanomacrophage progenitor cell	(107)
*Paralichthys olivaceus*	Spleen	FSP	Epitheloid	Not assessed	(108)
*Salmo salar*	Head kidney	SHK-1	Macrophage-like	Some macrophage-like properties	(109)
*Salmo salar*	Head kidney	SSP-9	Epitheloid	Constitutive and poly I:C inducible expression of immune-related genes	(110)
*Salmo salar*	Head kidney	TO	Dendritic-like	High phagocytic activity, no respiratory burst activity, LPS-inducible immune gene expression, no M-CSFR marker but CD83	(111)
*Scophthalmus maximus*	Trunk kidney	TK	Fibroblast-like	Not assessed	(112)

Synonymous terms to head kidney: pronephros, anterior kidney; synonymous terms to trunk kidney: mesonephros, posterior kidney.

Genetically modified fish cell lines have been employed to study the function of immune genes and their role in resistance of fish to pathogens (115), but they have not yet been applied for immunotoxicity testing. Ecotoxicological screening batteries to characterize the toxicity profile of chemicals or environmental samples have occasionally included immune-related reporter systems (116), however, those systems were based on either yeast or mammalian cells, but not on fish cells. In addition, the measured endpoints like NFκB signalling were selected as indicators of cellular stress rather than as immunotoxicity endpoint.

While *in vitro* systems have been developed for phagocytic and lymphocytic cells of the fish immune system, little attention has been given to the antigen-presenting dendritic cells which bridge innate and adaptive immunity. An early attempt was made by Ganassin and Bols (117) who established long-term rainbow trout spleen cultures which produced cells displaying the morphology and motility typical of dendritic cells. Bassity and Clark (118), adapting mammalian protocols for the generation of dendritic cells, succeeded in culturing non-adherent cells, which were classified as dendritic cells because of their motility, tree-like morphology, phagocytotic abilities and the expression of dendritic cell markers. Also Pettersen et al. (111) succeeded in establishing a fish cell line with dendritic-like properties. However, all these systems have not yet been applied for immunotoxicity studies.

### Primary Immune Cells of Fish

**Figure 1:**
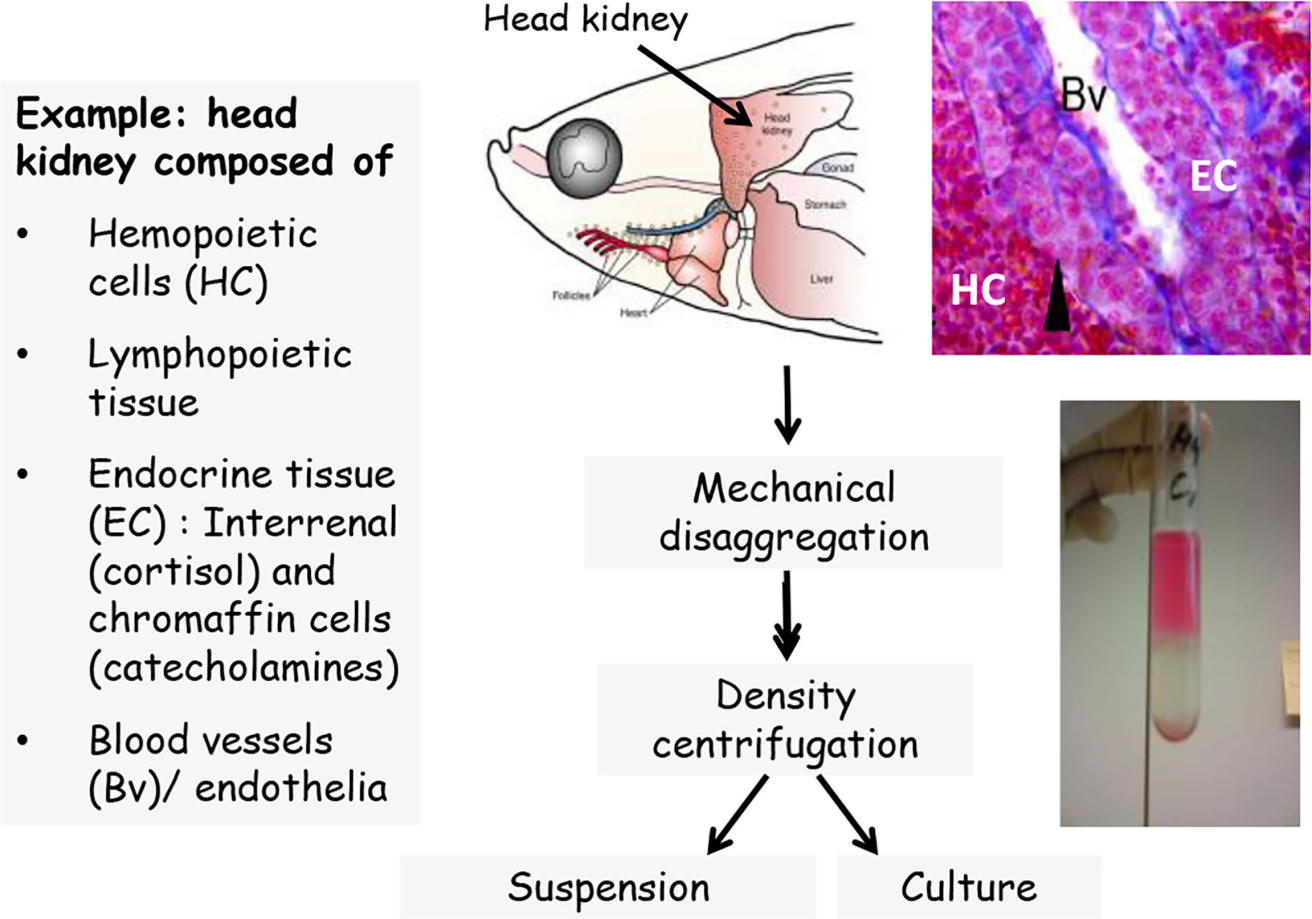
Scheme of leukocyte isolation, exemplified for head kidney.

The leukocytes of fish include lymphocytes, polymorphonuclear granulocytes (e.g., neutrophils), mononuclear phagocytes (tissues macrophages and circulating monocytes), dendritic cells and natural killer cells (119, 120). The leukocytes differentiate from hematopoietic stem cells which give rise to the lymphoid and myeloid lineages (121). Methods for isolation of immune cells for *in vitro* studies are available for all immune organs of fish, including head kidney, trunk kidney, spleen, thymus, the lymphoid tissues in barrier organs like the gut as well as the blood and the peritoneal cavity [e.g. (122–129)]. The principal steps for isolating fish immune cells from lymphoid tissues involve the – usually mechanical – disaggregation of the organ, followed by density centrifugation and/or hypotonic lysis in order to separate the leukocytes from erythrocytes (**Figure 1**). Most density centrifugation methods used for immune cell isolation yield mixed leukocyte populations containing a variety of innate and adaptive immune cells. Subfractions enriched in specific immune cell types can be obtained through the choice of the density gradient used for the isolation step, or through separation steps during subsequent culture (79, 130, 131). For instance, innate and adaptive immune cells can be at least partly separated by culturing the cells overnight; then the phagocytes will attach to the culture plate while the lymphocytes will remain floating and can be washed away [e.g., (132, 133)]. Methods to characterize the composition of immune cell populations include cytochemical staining, immunostaining, flow cytometry or cell sorting [e.g., (61, 134–138)]. Importantly, the isolation method can influence the performance of the isolated cells (128).

After isolation and separation, the immune cells are maintained *in vitro* as suspension for a few hours, or they are cultured up to several days or weeks, either in suspension, as monolayer or as three-dimensional aggregate [e.g., (79, 127, 139–142)]. During culture, various factors like cell density, cell composition or medium composition influence the performance of the cells (128, 131, 142, 143). For instance, supplementation of the culture media with arginine or glutamine significantly enhanced the mitogenic response of naive T- and B-cells of channel catfish (144). Media factors can also trigger the further differentiation of cultured immune cells, for instance, the differentiation of head kidney leukocytes into specific macrophage sub-populations, (145, 146), or the polarization of macrophages into inflammatory M1 macrophages and anti-inflammatory M2 macrophages (147).

## Application of Fish Cell-Based *In Vitro* Systems for Immunotoxicity Studies

Several reviews described basic principles of the piscine immune system, together with a discussion of the possible impacts of chemicals on fish immunity as well as the methodological approaches to test for immunotoxicity (26, 28–30, 32, 48). Here we focus on the use of *in vitro* assays in fish immunotoxicity assessment. As said above, a distinction must be made between *ex vivo* and *in vitro* approaches (32, 61, 79). In the first case, fishes are exposed *in vivo*, but afterwards the immune cells are isolated and their performance is studied *in vitro*. This approach reveals whether the *in vivo* exposure had consequences for the functional performance of the immune cells. The second approach is entirely *in vitro*. In the case of primary cells (see below), this means that the cells are isolated from control fish that have not been exposed to the toxicant. Still, *in vivo* factors like the nutritional status or the sex of the donor fish may influence the *in vitro* performance of the isolated immune cells [e.g., (148)]. Also the circadian time point of cell isolation may have an influence on the performance of the isolated cells (149).

A tool that might be of use for fish immunotoxicity assessment are fish embryos. Tests with embryonic life stages of fish are not considered as animal tests by law, and are increasingly used as alternatives to *in vivo* fish tests in the sense of the 3R (reduce, replace, refine) concept (150). Embryonic life stages of fishes have an at least partly functional immune system, for instance, in zebrafish, the innate immune system differentiates in the course of this developmental period (151, 152). Zebrafish embryos were extensively used as model organisms to study vertebrate hematopoietic development [e.g., (153)]. On that basis, zebrafish embryos may be well suitable as test system for assessing myelotoxic effects of chemicals in fish, however, this potential has been rarely used to date (154). In the present communication, we will not include fish embryos as immunotoxicity test systems, since we will strictly focus on *in vitro* methodologies.

Chemicals impact the immune system through direct interactions with immune cell survival, proliferation and functioning, and with the immune system communication. As a result, immunocompetence may get compromised (immunosuppression), leading to increased risk of infection and cancer. Alternatively, the toxic impact may cause immunostimulation, which can result in hypersensitivity, allergy or autoimmune reactions. Immunotoxicological research on fish focused to date almost exclusively on immunosuppressive effects, whereas immunostimulation, which is a frequent response of mammalian immunity to toxic exposure, either plays no role or has not been sufficiently studied in fish (32). Chemicals may also indirectly modulate the immune system, for instance, the costs incurred by the defense activities against the chemicals may trade-off with resource allocation to the maintenance and activation of the immune system. Further indirect effects of toxic chemicals on immunity can arise, for instance, when the chemicals cause cell damage and cell death in non-immune tissues, leading to the release of DAMPs (damage-associated molecular patterns) which then activate specific receptors on immune cells and trigger an immune responses (155).

**Figure 2:**
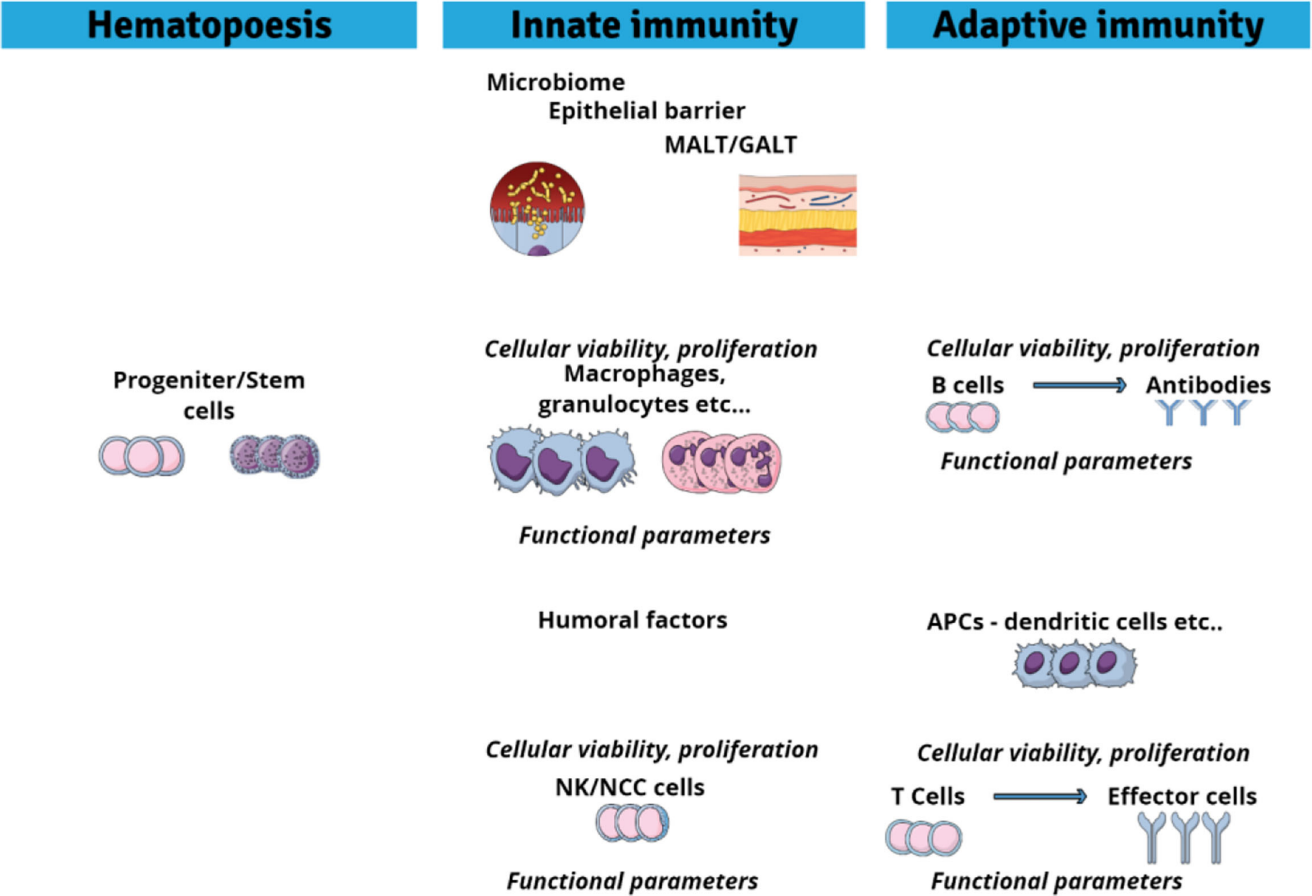
Potential cellular targets sites for immunotoxic agents.

*In vitro* assays can detect direct chemical effects on cell viability, differentiation and proliferation as well as on cell functions (64, 68). The main potential immune cell targets are summarized in **Figure 2**. In the following we will discuss which *in vitro* cell systems and endpoints are available to assess immunotoxic activities of chemicals in fish. While a number of studies assessed immune endpoints, particularly immune gene expression, in non-immune cells of fish (90, 156–159), this review will focus on studies that employed immune cells. The vast majority of toxicological studies with fish immune cells were done using primary immune cells, while cell lines were rarely used (see also **Table 2**).

**Table 2:** Examples of *in vitro* immunotoxicity studies using fish immune cells.

Species	Immune cell type/tissue of origin	Toxic agent(s)	Experimental setting	Endpoints and effects	Reference
**Heavy metals and organometals**					
*Oncorhynchus mykiss*	Phagocytes from head kidney (primary cells)	Cu^2+^ Al^2+^ Cd^2+^	Exposure to various concentrations before, 1 hr or 24 hrs after treatment with PHA or bacteria	Respiratory burst activity was decreased or increased, depending on the timepoint/duration of exposure	(160)
*Oncorhynchus mykiss*	Blood and head kidney leukocytes (primary cells)	Hg^2+,^ methyl-Hg	*in vitro* exposure to various concentrations in presence/absence of PHA, ConA, LPS	Only close-to-cytotoxic concentrations had an effect on phagocytosis, respiratory burst activity, and mitogenic response	(161)
*Ictalurus melas*	Natural Killer (NK) cells from head kidney (primary cells)	Cd^2+^	Co-exposure to Cd^2+^ and target cells (K-562 human cell line) at 26°C	Concentration-dependent decrease of the cytotoxic activity of the NK cells	(162)
*Cyprinus carpio*	leukocytes from head kidney (primary cells)	Zn^2+^ Mn^2+^	Exposure to various concentrations together with mitogen (PHA, Con A, LPS)	Concentration-dependent decrease of mitogen-stimulated cell proliferation by Zn^2+^, whereas the Mn^2+^ effect varied with metal concentration, exposure timing, and type of mitogen	(163)
*Cyprinus carpio*	Leukocytes from blood and head kidney (primary cells)	Cr(VI)	Exposure for 2 to 6 days to various concentrations of Cr^2+^, partly in presence of pokeweed mitogen at 25°C	Immune effects occurred at sub-cytotoxic concentrations; Concentration-dependent decrease of nitric oxide production, respiratory burst activity, and mitogen-stimulated cell proliferation	(164)
*Dicentrachus labrax*	Leukocytes from head kidney (primary cells)	Cd^2+^ Hg^2+^ As^2+^ Pb^2+^ Methyl-Hg	30-min-exposure to various concentrations at 25°C	Concentration-dependent effects on apoptosis and partly on necrosis. All metals induced decrease of phagocytosis. Effect on respiratory burst activity varied with metal concentration; increasing effect on immune gene expression	(165)
*Opsanus tau, Trinectes maculates, Micropogonias undulatus*	Leukocytes from kidney (primary cells)	Tributyltin TBT	Exposure to increasing concentrations up to 18 hrs at 25°C	Concentration-dependent decrease of phagocytosis, with *Micropogonias undulatus* being the most sensitive species. Suppressive effect increased with exposure duration	(166)
*Oncorhynchus mykiss*	Leukocytes from spleen and head kidney (primary cells)	Tributyltin TBT Dibutyltin DBT	Exposure to increasing TBT and DBT concentrations up to 7 days at 15°C	Concentration-dependent decrease of mitogen-stimulated cell proliferation, whereas cytotoxic natural killer cell activity was not inhibited. Spleen leukocytes were more sensitive than head kidney leukocytes	(167)
*Melanotaenia fluviatilis, Bidyanus bidyanus, Macquaria ambigua Macculochella peeli*	Leukocytes from head kidney (primary cells)	Tributyltin TBT Dibutyltin DBT	Exposure to increasing TBT and DBT concentrations up to 48 hrs at 22°C	Concentration-dependent decrease of phagocytosis, with species differences in sensitivity	(168)
**Pesticides**					
*Melanotaenia fluviatilis, Bidyanus bidyanus, Macquaria ambigua Macculochella peeli*	Leukocytes from head kidney (primary cells)	Endosulfan, chlorpyrifos	Exposure to increasing concentrations up to 48 hrs at 22°C	Endosulfan caused a moderate concentration-dependent decrease of phagocytosis in all species except *Bidyanus bidyanus*; chlorpyrifos had a moderate effect on phagocytosis in *Maccullochella peeli*.	(168)
*Oncorhynchus mykiss*	Leukocytes from peripheral blood (primary cells)	Atrazin, permethrin, piperonyl butoxide	Exposure to increasing concentrations for 96 hrs at 15°C	Concentration-dependent decrease of cell viability and mitogen-stimulated proliferation	(169)
*Oncorhynchus tshawytscha*	Leukocytes from spleen and head kidney (primary cells)	p,p’-DDE	Exposure to increasing concentrations up to 50 hrs at 17°C	Concentration-and time-dependent decrease of cell viability and mitogen-stimulated cell proliferation. Head kidney leukocytes were more sensitive than spleen leukocytes. Leukocyte responses differed seasonally.	(170)
*Sparus aurata*	Leukocytes from head kidney (primary cells)	p,p’-DDE lindane	Exposure to increasing concentrations for 24 hrs at 22°C	No effect on cell viability and only slight effects on phagocytosis, respiratory burst activity and cell-mediated cytotoxicity. Upregulation of a number of immune genes	(171)
*Morone saxatilus*	Leukocytes from head kidney (primary cells)	Chlorothalonil	Exposure to increasing concentrations for 20 hrs at 21-23°C	Concentration-dependent decrease respiratory burst activity but not of phagocytosis	(172)
*Oreochromis niloticus*	Leukocytes from spleen (primary cells)	Endosulfan	Exposure to one concentration of endosulfan up to 72h at 28°C	Endosulfan per se increased lymphoproliferation but decreased mitogen-stimulated proliferation	(173)
**Polyaromatic hydrocarbons (PAHs)**					
*Cyprinus carpio*	Leukocytes from head kidney (primary cells)	3-methyl-cholanthrene	Exposure to increasing concentrations for 1 h at 21°C	Concentration-dependent increase of respiratory burst activity in PMA-stimulated leukocytes bot not in unstimulated leukocytes.	(132)
*Cyprinus carpio*	Leukocytes from head kidney (primary cells)	3-methyl-cholanthrene	Exposure to increasing concentrations for 72 hrs at 28°C	Concentration-independent inhibition of ConA- or LPS-stimulated lymphocyte proliferation	(174)
**Poylchlorinated biphenyls (PCBs)**					
*Salvelinus namaycush*	Thymocytes from thymus (primary cells)	Aroclor 1254	Exposure to increasing concentrations up to 24 hrs at 17°C, alone or in combination with LPS or cortisol	Concentration-dependent increase of apoptosis and necrosis. LPS had no effect on toxicity, but cortisol enhanced the toxicity	(175 176)
*Oncorhynchus mykiss*	Leukocytes from head kidney (primary cells)	PCB 126	Exposure to increasing concentrations up to 24 hrs at 22°C, with or without cortisol pre-incubation	Concentration-dependent, transient increase of IL-1β expression. Pre-incubation with cortisol decreased the PCB 126-induced IL1-β expression	(177)
**Endocrine-disrupting compounds**					
*Sparus aurata*	Leukocytes from head kidney (primary cells)	Ethinylestradiol (EE2)	Exposure to increasing concentrations up to 48 hrs, in the presence or absence of bacterial DNA (VaDNA)	No effect on leukocyte viability. Concentration-dependent decrease of phagocytosis. Respiratory burst activity was not altered by EE2 alone, but inhibition of the VaDNA-induced respiratory burst activity. Time- and concentration-dependent complex effects on immune gene expression.	(178)
*Cyprinus carpio*	Leukocytes from head kidney (primary cells)	Bisphenol A (BPA), nonylphenol	Exposure to increasing concentrations for 10 hrs at 20°C	Both compounds induced a concentration-dependent increase of respiratory burst activity and a decrease of phagocytosis, while nitric oxide production was unchanged	(179)
*Cyprinus carpio*	Leukocytes from head kidney (primary cells)	Bisphenol A (BPA)	Exposure to increasing concentrations for 6 hrs at 26°C	No effect on leukocyte viability. Bactericidal and lysozyme activity were altered in an inverse U-shaped concentration-response curve. Concentration-dependent induction of NO production, of respiratory burst activity and of several immune genes like hepcidin, IL-10 and IL-1β	(180)
*Carassius auratus*	macrophage cells GMCL (cell line)	Estradiol (E2)	Exposure to increasing concentrations for 8 hrs at 20°C	Concentration-dependent inhibition of chemotaxis, but no effect on phagocytosis and nitric oxide production	(181)
*Carassius auratus*	Lympho-cytes and macro-phages from blood (primary cells)	Bisphenol A (BPA)	Exposure to increasing concentrations up to 24 hrs at 18°C	Concentration-dependent decrease of respiratory burst, and alteration of mitogen-stimulated cell proliferation.	(182)
**Pharmaceuticals**					
*Cyprinus carpio*	Leukocytes from head kidney (primary cells)	Amitriptyline, fluoxetine, mianserin (anti-depressants)	Exposure to increasing concentrations for 6 hrs at 26°C	Concentration-dependent decrease on bactericidal activity, respiratory burst, NO prodiction, NO synthase activity and pro-inflammatory cytokine expression. Stimulation of anti-inflammatory IL-10 expression	(183)
**Complex samples**					
*Oncorhynchus mykiss*	Leukocytes from head kidney (primary cells)	Solid phase extracts of 12 municipal effluents subjected to different treatment processes	Exposure to increasing extract concentrations for 24 hrs at 15°C	About half of the effluents decreased cell viability, 4 effluents decreased phagocytosis, 8 effluents increased phagocytosis	(184)
**Nanomaterials**					
*Cyprinus carpio*	Carp leukocyte cell line CLC (cell line)	Carbon nanofibers, graphene oxide	Exposure to increasing concentrations for 24 and 72 hrs at 30°C	Concentration-dependent decrease of cell viability. Uptake of nanomaterials	(185)

PHA, phytohaemagglutinin; Con A, concanavalin A; LPS, lipopolysaccharide; PMA, phorbol-12-myristate-13-acetate; NO, nitric oxide; ROS, reactive oxygen species.

### *In Vitro* Assays Related to Hematopoietic Progenitor Cells

Chemicals can cause a suppression in the production of progenitor cells that will differentiate into leukocytes, erythrocytes or thrombocytes. In mammals, this effect takes place in the bone marrow and is designated as bone marrow suppression or myelotoxicity. For the *in vitro* evaluation of myelotoxic activities of chemicals, bone marrow culture systems have been established which enable to assess proliferation and differentiation of pluripotent hematopoietic stem cells or of progenitors of specific blood cell lineages (186). An example is provided by the Colony-Forming Unit Granulocyte-Macrophage (CFU-GM) assay, which quantifies the number of surviving bone marrow progenitors as a function of chemical concentration (64, 186). Assessment of myelotoxicity is used as first step in a tiered *in vitro* immunotoxicity testing; if a chemical is found to be myelotoxic, this means that the organisms will no longer be able to produce immune cells and sustain a functional immune system. Therefore a further testing is no longer necessary (68).

In fish, the main hematopoietic organs include head kidney, spleen, and trunk kidney, their relative importance varying across species (121). Primary culture systems that support the proliferation and differentiation of piscine hematopoietic stem cells have been established from spleen and kidney (79, 117, 187–189). Although these systems hold promise for immunotoxicological studies, they have not been used yet for this purpose.

### *In Vitro* Assays Related to Epithelial Barrier Immunity

Mucosal epithelia establish a barrier between the environment and the internal milieu of the organism. Regulation of immunity at the barrier epithelia is critical to preserving host integrity. It is here where the immune cells and the mucosa-associated lymphoid tissues (MALT) are initially exposed to external factors including toxicants. Mucosal epithelia also harbour microbial communities, the microbiome, which influence mucosal homeostasis and immune function. Immune responses of epithelial barriers involve an intricate network of molecular pathways, epithelial cells, intraepithelial immune cells as well as the microbiome (190–192).

Toxicants can impair the epithelial barrier immunity of fish (27, 193–195). The challenge in using *in vitro* systems for studying toxic effects on mucosal immunity is that they have to reproduce the integration and interaction of immune processes as they occur the *in vivo* setting. Most *in vitro* models lack organ architecture, thereby reducing the possibilities for cell-cell interactions and interactions between the different components driving the immune response. Several approaches were developed over recent years to overcome these difficulties. Typically, cell lines such as the rainbow trout RTgutGC line are cultured on semipermeable supports in order to establish tight, polarized epithelia (196–198). The realism of these systems can be improved by combining different cell lines, by modifying the culture environment or by including microbial communities (199, 200). These *in vitro* systems have been successfully used to study the impact of toxicants on mucosal biotransformation and oxidative stress pathways (157, 158). In addition, they are responsive to immune stimuli such as lipopolysaccharide, and are able to upregulate pro-inflammatory cytokines (159). A logical next step could be, analogous to what is done with *in vitro* models of mammalian mucosal barriers [e.g. (201)], to include immunocompetent cells into the epithelial layers. Overall, three-dimensional *in vitro* systems, particularly when incorporating immune cells, hold promise as tools for studying xenobiotic effects on mucosal immunity of fish.

### Phagocyte-Based *In Vitro* Assays

The blood cells of teleost fish consist of erythrocytes (> 90%), thrombocytes and leukocytes (120). Fish thrombocytes may exert leukocyte-like functions, but this is discussed controversially (120, 202). Since they have not been used for *in vitro* immunotoxicity assays, we will not further consider them here.

Phagocytic cells of the innate arm of the teleostean immune system arise from myeloid stem cells and include monocytes/macrophages and granulocytes (eosinophilic, basophilic, neutrophil) (26, 28, 30, 120, 203). Granulocytes and monocytes/macrophages migrate to pathogens, and incorporate and kill them. They are present in the peripheral blood from where they can invade (injured) tissues. Monocytes, after migrating out of the blood into tissues, differentiate into macrophages. There exist also resident phagocytic cell populations, for instance, microglial cells in the brain or Kupffer cells in the liver, which form the so-called reticuloendothelial system (RES) (28). The majority of *in vitro* fish immunotoxicity studies used cells isolated from the head kidney and the peripheral blood (129). Importantly, the isolated cell populations may show significant functional differences, depending on their cellular composition, their tissue of origin, the physiology of the donor fish, and/or the culture microenvironment (120). Also cell lines with properties of phagocytic cell types are available, for instance, the goldfish macrophage cell line GMLC (181), or the monocyte-macrophage cell line RTS11 from rainbow trout (105), however, innate immune cell lines have been rarely applied in immunotoxicity studies. The most frequently used *in vitro* system to investigate chemical impacts on innate immune functions of fish are freshly isolated or cultured phagocytes. As pointed out by Fournier et al. (204) and Bols et al. (79), phagocytes have properties especially useful in the context of ecotoxicology. Phagocytosis is conserved in all animals what allows cross-species comparisons. Often phagocytes can be collected by non-lethal techniques. In the intact animal, exposure of phagocytes to xenobiotics is assured because phagocyte populations are found at all potential site of xenobiotic entry (gills, gut, skin). Because of their capability for pino- and phagocytosis, phagocytes can take up not only dissolved but also particulate foreign materials and protein-bound chemicals. Finally, phagocytes are able to metabolize xenobiotics (135, 205).

The fish phagocyte populations used for *in vitro* studies are often of mixed composition, i.e. they contain hematopietic precursor cells, monocytes/macrophages, and various types of granulocytes. For instance, Ribas et al. (206) reported that a phagocyte population isolated from the head kidney of the freshwater fish, *Hoplias malabaricus*, was composed of 71% hematopoietic precursor cells, 19% macrophages and 9% monocytes. The endpoints most frequently measured in *in vitro* toxicity studies with fish immune cells include chemical effects on cell viability, phagocytotic activity, respiratory burst activity and immune gene expression (32, 79, 129).

*Cytotoxicity* is commonly understood in the sense that the cell is killed by the chemical agent. If the chemical perturbs the metabolic or structural integrity of the cell, this can cause cell death. Toxicants can also trigger physiological cell death, i.e., apoptosis, as it has been shown, for instance, for organochlorine contaminants and polyaromatic hydrocarbons (PAHs) (175, 207). Methods to determine cytotoxicity are principally based on (i) the assessment of cell membrane integrity, e.g., by determining the exclusion of dyes such as trypan blue, (ii) the retainment of intracellular components such as cytoplasmic enzymes, or (iii) the measurement of the cellular metabolic activity (78, 208). In numerous *in vitro* fish immunotoxicological studies, such methods have been applied to evaluate at which concentration the test chemical causes the death of the immune cells [e.g., (161, 165, 172, 209, 210), see also **Table 2**]. It is important to distinguish between the cytotoxic and concentration of a test chemical and the concentration at which it causes specific immune functional effects, e.g., altered phagocytotic activity. The cytotoxic concentration of a test chemical activates numerous non-specific defense and lethality mechanisms. This concentration represents no specific immunotoxic activity, but general baseline cytotoxicity (208, 211, 212). To test for specific immunotoxic effects *in vitro*, it is essential to apply non-cytotoxic concentrations in order to avoid false positive responses caused by the interference of the cytotoxic concentrations with cell viability (213, 214).

*Phagocytosis* is a non-specific immune function which refers to the cellular uptake and intracellular processing of pathogens, foreign particles, cellular debris and macromolecules. Among immune cells, particularly cells of the innate arm such as monocytes, macrophages and granulocytes possess phagocytic capabilities, but in fish also B cells and thrombocytes display phagocytic activities (202, 215–217). The analysis of chemical-induced suppression of phagocytosis is of toxicological relevance as it can disturb the clearance of pathogens, the processing and presentation of antigens as well as cytokine secretion and immune system communication, what may result in a compromised immunocompetence of the organism. Methodologically, phagocytotic activity is determined by measuring the ingestion of (inactivated) bacteria or plastic beads. Often these materials are fluorescently labelled so that their uptake can be monitored by flow cytometry, in fluorescent plate readers, or by fluorescence microscopy (129, 218–221). Assay conditions such as incubation time or particle concentration, the analytical method as well as whether the phagocytes are activated or resting influence the results of phagocytosis assays (184, 222). Also the use of appropriate controls to distinguish between particles adhered to the surface of the cells or ingested by the cells is mandatory (214, 217). Importantly, toxicant effects on phagocytosis may be detectable only after activation of the cells by exposure to pathogenic signals such as bacterial lipopolysaccharide (LPS); the activation can lead to enhanced phagocytosis, enhanced production of reactive oxygen intermediates and nitric oxide, and enhanced secretion of pro-inflammatory cytokines (IL-1 TNFa, IL-6, IL-12, IL-1 [e.g., (141, 214, 223, 224)]. The results of the phagocytosis assay are presented as percentage of phagocytosing cells in the cell population, or as number of particles engulfed per cell (26, 61, 184, 225). This assay provides a simple assessment of an important (innate) immune mechanism, and consequently, it has been frequently used for *in vitro* immunotoxicity studies with innate immune cells of fish. Diverse groups of chemicals as well as natural toxins and micro/nanoparticles have been shown to modulate the phagocytic activity of the cells. Generally a trend for suppression of phagocytosis has been observed in *in vitro* immunotoxicity studies with innate immune cells of fish; only rarely a stimulation has been reported (226). Remarkably, the *in vitro* assays appear to reflect species as well as sex differences in the phagocytic response to toxicants (168, 227).

*Respiratory or oxidative burst* by phagocytes involves the production of reactive oxygen species (ROS) as well as nitrogen radicals (NO). The ROS reaction is catalysed by a NADPH oxidase complex (228, 229). The radicals produced by the respiratory burst serve to kill invading microorganisms, either extracellularly, or intracellularly after phagocytosis, and thus respiratory burst has an important role in the immune response towards pathogens (230). In studies with fish leukocytes, frequently used methods to measure the respiratory burst activity are the reduction of the dye nitroblue tetrazolium, and the luminal-enhanced chemoluminescence (27, 61, 79, 231). Various stimulants have been used to induce phagocyte respiratory burst, including pathogen-derived PAMPs, or agents such as concavalin A, zymosan phorbol myristate acetate (PMA), with the choice of the stimulating agent being of relevance for the response of the cells against pathogens and toxicants (27, 61, 129, 232–234). Analysis of the respiratory burst activity has been frequently applied in immunotoxicity studies with isolated fish phagocytes (see **Table 2**). While for phagocytosis activity, generally suppressive effects of toxicants have been reported, for respiratory burst activity also chemical-induced activation was observed, particularly for estrogenic endocrine disruptors, but the effects vary largely with exposure conditions and whether the immune cells are resting or stimulated cells (cf. **Table 2**). For instance, *in vitro* exposure to Ni^2+^ did not affect the respiratory burst activity of activated peritoneal macrophages of rainbow trout, but it affected the basal ROS production of these cells (235). Also biological factors such as species differences influence the results of the *in vitro* studies (27, 236) as well as the purity of the test agent (237). Toxicants can also potentiate the induction of phagocyte ROS production by stimulants such as PMA, as it has been shown by Reynaud et al. (132) for the PAH, 3-methylcholanthrene (3MC). This potentiating effect could involve 3MC biotransformation – as phagocytes possess the capacity for xenobiotic metabolism (135) - and/or modulation of intracellular Ca levels. This example may illustrate the complexity of mechanisms through which chemicals can modulate phagocyte functions.

The examination of *immune gene expression* is increasingly used for *in vitro* studies on toxic effects on fish phagocytes, both with primary cells and with cell lines. Methodologically, RT-PCR methods for the measurement of individual genes and global transcriptomic analyses have been applied. As with phagocytosis and respiratory burst, transcriptomic responses of fish phagocytes are largely influenced by biological factors and experimental/technical settings. Mainly pro-inflammatory cytokines such as TNF-α, IL-1β, or IL-6 were studied, but also anti-inflammatory cytokines such as IL-10 (see **Table 2**). The transcriptomic analyses revealed the regulation of key immune signalling pathways such as NFκB, ERK1/2, Toll-like receptor, or B cell receptor by the toxicants, often together with cellular stress-related pathways like Jak-STAT (173, 180, 183, 238). Gene expression approaches were also applied for mechanistic studies, for instance, to unravel the role of the estrogen receptors (ERs) or the peroxisome proliferator-activated receptor,PPARγ, in mediating chemical effects on immune functions, or to study the interaction between toxicants and hormones of the stress axis in modulating immune gene expression (177, 180, 210, 239, 240).

Further endpoints that have been used, although relatively rarely, for *in vitro* immunotoxicity studies with piscine phagocytes include the assessment of phagocyte chemotaxis and bactericidal assays. In order to engulf and incorporate pathogens, phagocytes have the ability of chemotaxis, and this property appears to be sensitive to the action of toxicants (181, 241). Ni^2+^, for instance, altered the migration of rainbow trout macrophages *in vitro* (235). The bactericidal or bacterial killing assay measures the capacity of isolated phagocytes to kill bacteria. It has been applied, for instance, to examine the immunosuppressive effect of pentachlorophenol on phagocytes of *Fundulus heteroclitus* (242).

### Lymphocyte-Based *In Vitro* Assays

The lymphoid lineage of leukocytes includes B-lymphocytes, natural killer (NK) cells and T lymphocytes, the latter being composed of cytotoxic T cells, T helper cells and regulatory T cells (26, 28, 30, 243–245). The T cells of teleost fish display gene expression patterns that resemble the T cell subpopulations known from mammals, namely cytotoxic (CD8), helper (CD4) and regulatory (Treg) T cells (203, 246). Also teleost B cells are composed of diverse subsets, and express different heavy Ig chain classes, including IgM, Ig T/Z, and IgD (247, 248). NK cells, like the cytotoxic T cells, initiate the killing of altered, tumorous and infected cells, but different to cytotoxic T cells, they do not depend on specific antigen presentation for recognizing infected cells. When NK cells were first described in fish, they were designated as non-specific cytotoxic cells (NCC). While the NK cells belong to the innate arm of the immune system, the cytotoxic T cells belong to the adaptive arm. Evolutionary-wise, there appears to be a homology between teleost lymphocytes and mammalian innate-like lymphocytes (249).

To assess immunotoxic effects on fish lymphocytes *in vitro*, typically no purified or enriched lymphocyte cultures are used, but mixed leukocyte cultures, which are then treated under conditions that elicit B or T cell-specific responses. An example is provided by the widely used *lymphoproliferation* or lymphocyte blastogenesis assay. When lymphocytes are challenged with a pathogen, they undergo proliferation. The immunocompetence of a fish will be compromised if a toxic agent suppresses the functional ability of lymphocytes to proliferate. To assess whether a chemical can inhibit lymphocyte proliferation, leukocytes isolated from blood or from lymphoid organs are exposed during *in vitro* culture to mitogens, and the magnitude of cell proliferation is then measured as endpoint. Commonly used mitogens for T cells include the plant lectins, phytohaemagglutinin (PHA) and concanavalin A, while for B cells, lipopolysaccharide (LPS) from the wall of gram-negative bacteria is used, and pokeweed mitogen serves as mixed mitogen for B and T cells (79). Methods to measure the induced cell proliferation include the DNA-incorporation of radiolabeld thymidine or thymidine analogues, the determination of the cells undergoing mitosis (mitotic index), or colorimetric methods (61, 250–252). Also flow cytometry is a frequently used method (253), also because it provides the option to determine the proliferation of specific lymphocyte subpopulations (254). The proliferative response obtained in *in vitro* blastogenesis assays varies with a number of factors including the mitogen type and concentration, the length of incubation time or the composition of the culture medium (79). The lymphoproliferation assay has been widely applied in the immunotoxicity assessment of diverse compounds including metals, PAHs, endocrine disruptors or natural toxins [(163, 172, 174, 182, 255–259), see also **Table 2**].

As an indicator of the functionality of the antibody producing B cells, the plaque forming cell (PFC) assay can be used (260). Although the plaque formation is determined *in vitro*, the assay is, strictly speaking, an *ex vivo* assay, since the fish has to be injected with the antigen – mostly sheep red blood cells - *in vivo*. The toxicant exposure is usually also done *in vivo*. Only occasionally the assay is performed fully *in vitro* (261), but to the best our knowledge, this approach has not been used for toxicity studies. Since it is not an entirely *in vitro* test, the PFC assay will not be discussed here.

*In vitro* approaches were also used to study toxic impacts of chemicals on thymocyte precursor cells. Sweet et al. (175) found that exposure of isolated thymic cells of Lake trout (Salvelinus namaycush) to various organochlorine contaminants (Aroclor 1254, hexachlorocyclohexane isomers) displayed high levels of apoptosis. Subsequent studies confirmed these findings and showed that the presence of cortisol enhanced the chemical toxicity (176).

NK cells and cytotoxic T cells are responsible for the cell-mediated cytotoxicity (CMC). For various *in vitro* preparations of fish immune cells, an allogeneic and xenogeneic activity has been demonstrated. These preparations contained cells expressing markers of NK cells and/or cytotoxic T cells (cf. 203). For NK cells, methods for their isolation and primary culture have been developed (79, 262). While a number of studies used *ex vivo* approaches to investigate toxic effects on fish NK cells [e.g., (263)], toxic effects on NK cells have been very rarely studied using *in vitro* systems. An example is the work of Viola et al. (162) who showed that Cd had an inhibitory effect on the ability of catfish NK cells to kill foreign cells of a human cell line.

## Perspectives in Using *In Vitro* Systems for Immunotoxicity Assessment With Fishes

The growing evidence that diverse environmental contaminants including pharmaceuticals, endocrine disruptors, polyaromatic hydrocarbons, organochlorines, or plastic materials can interfere with immune functioning of fish argues for a consideration of immunotoxicity in the ecotoxicological hazard assessment. However, as recently pointed out by Johnson et al. (2) adding more and more tests to the existing battery of the Organisation of Economic Cooperation and Development (OECD) battery of ecotoxicity tests is not realistic, for both practical and ethical reasons. Here, mode of action- and mechanism-oriented screening and toxicity profiling strategies increasingly rely on *in vitro* assays as rapid, non-animal and cost-effective tools, as exemplified by the US EPA ToxCast Program for monosubstancess [e.g., (213, 264)] or by bioanalytical approaches for complex environmental samples [e.g., (52, 265)]. Such approaches could well integrate *in vitro* assays for immunotoxicity screening. In fact, the ToxCast Programme includes already a number of human cell-based assays that express genes or proteins of the innate and adaptive immune responses, although functional immune endpoints are not yet included (266). Here, we aimed to evaluate the availability and potential of fish-based *in vitro* assays to screen for immunotoxic activities of environmental agents to fish.

### *In Vitro* Assays With Fish Immune Cells for Mechanistic Studies

*In vitro* systems are well suitable to investigate the mechanisms through which toxicants interfere with immune cell functions. This is illustrated, for instance, by studies on the interference of PAHs with Ca and cAMP signalling and biotransformation processes of fish immune cells (132, 135, 267), the influence of endocrine disruptors and pesticides on signal transduction pathways (180, 183, 240, 268), the interference of toxicants with cell differentiation processes (259), or by studies that examine whether immunotoxic effects are caused by a direct action of a chemical on the immune cells or whether they may be caused by indirect effects (269). Also toxicokinetic aspects such as determinants for binding and uptake of toxic agents by immune cells can well be examined using *in vitro* systems (222, 227). In addition, *in vitro* systems offer the possibility to compare immunotoxic processes at the cellular level under equivalent conditions across species (79, 168, 236). Likewise, *in vitro* systems provide the option to study cell type-specific responses to toxic agents, e.g., to compare phagocytes versus lymphocytes, to compare leukocytes originating from different immune organs, or to compare immune cells versus stromal cells (141, 182, 270). Finally, the role of the physiological condition of the donor fish in the immune cell response can be detected. For instance, Ottinger and Kaattari (271) showed that the sensitivity of rainbow trout leukocytes to aflatoxin varied with the season, i.e. cells prepared from fish during January to June were significantly less sensitive than those from July to December.

### *In Vitro* Assays With Fish Immunce Cells to Screen for Immunotoxic Activities of Environmental Agents

The relatively few *in vitro* toxicity studies with fish immune cells are dispersed between toxicant classes, toxic modes of action, immune cell types, immune endpoints, fish species and assay conditions. For instance, for the group of arylhydrocarbon receptor (AhR)-binding PCBs, which are well known immunotoxicants impacting the health and disease status of wild fish populations [cf. (272)], there is fairly good number of *ex vivo* studies available, however, to the best of our knowledge only six studies have investigated the PCB effects on fish immune cells *in vitro*. Quabius et al. (177) exposed primary cultures of rainbow trout anterior kidney cells to the AhR-binding PCB 126, and observed a transient, but significant induction of the cytokine IL-1β. The induction was potentiated by the presence of LPS. Zhang et al. (273, 274) exposed isolated *Carassius auratus* lymphocytes to PCBs and found that the EC50 for apoptosis was higher for higher chlorine substitution, and for coplanar than for non-planar structure. Vazzana et al. (275) treated isolated lymphocytes of sharpsnout sea bream, *Diplodus puntazzo*, with Aroclor 1254, a PCB mixture, and observed an enhanced respiratory burst activity of the cells. Sweet et al. (175) and Miller et al. (176) exposed thymocytes of Lake trout to Aroclor 1254 and found that this resulted in a significant increase of thymocyte apoptosis. Such a data set, while valuable, is too fragmented and limited to come up with an at least partly conclusive immunotoxicity profile of AhR-binding PCBs in fish, or recommendations for the most appropriate assay for PCB immunotoxicity screening.

Further factors that currently limit the utility of piscine *in vitro* assays for immunotoxicity screening include the lack of assay standardization (79) as well as the still limited repertoire of *in vitro* immunoassays. Currently there exist no fish correlates for important screening assays as they are frequently used in human immunotoxicology, for instance, myelotoxicity assays, the NK killing assay, fluorescent cell chip assays or assays assessing multiple immune endpoints [cf. (60, 65, 68)].

A critical question in the use of in pisicine vitro assays for immunotoxicity screening is whether they correctly classify test agents as potentially immune-active or –inactive, i.e. if they produce false positives or false negatives. Rehberger et al. (214) tested five immunotoxic chemicals and two non-immunotoxic chemicals at sub-cytotoxic concentrations using an *in vitro* assay with head kidney leukocytes of rainbow trout. The five immunotoxicants were correctly classified as immunotoxicants, although the pharmaceutical diclofenac elicited a rather weak response (pointing to the need of using a test battery, since depending on the mode of immunotoxic action, different *in vitro* assays will show different sensitivities). However, the two non-immunotoxic chemicals, displaying a narcotic mode of action – butanol and ethylene glycol – also induced an immune response. This false positive result may be explained by an overlap of the cellular immune response with the cellular stress response. The two cellular responses share a number of receptors and signalling pathways (276), many of them being located or associated with the cell membrane. Chemicals with narcotic mode of action interfere with the cell membrane organization and fluidity, and this activates stress pathways (277) which then may converge with immune-related receptors and signalling pathways (214). If the hypothesis of an interference between the cellular stress and immune responses is correct, this would represent a principal obstacle in using cell-based assays for *in vitro* immunotoxicity screening.

### *In Vitro* Assays With Fish Immunce Cells to Predict *In Vivo* Immunotoxicity in Fish

The discussion on false positives and negative results leads to the question how (qualitatively) predictive the *in vitro* results are for the *in vivo* immunotoxic action of environmental agents. Again, the available fragmentary database makes it difficult to provide a conclusive answer. For instance, the *in vitro* findings on the apoptotic effects of PCBs on fish thymocytes do well agree with the established suppressive effect of PCBs on the fish thymus *in vivo* (cf. 272). In contrast, the *in vitro* observations that PCBs induce the respiratory burst activity of leukocytes (273–275) is not in line with reports that *in vivo* PCB exposure of fish results in the suppression of the respiratory burst activity (263, 278). Rehberger et al. (32) performed a meta-analysis of published *in vitro* and *in vivo* immunotoxicological studies with fishes and found some correlations but also numerous misfits. The authors emphasized that the robustness of the correlations across the different studies was weak due to the low number of data points. Also when analysing only those publications that included a direct *in vivo-in vitro* comparison within the same study, for instance, the study of Cabas et al. (178) who compared the immunological effects of an estrogen-active endocrine disruptor in gilthead seabream (*Sparus aurata*) *in vivo* and in the isolated head kidney leukocytes *in vitro*, a conclusive answer was not possible because the number of studies was too low.

In human toxicology, the regulatory assessment of immunotoxicity still exclusively relies on *in vivo* tests, although the use of *in vitro* approaches for the prediction of direct immunosuppressive effect is increasingly discussed (68, 186, 279). A decision tree approach has been suggested for the *in vitro* assessment of chemical-induced immunosuppression which combines different cell systems and endpoints in a tiered manner (60, 68). Another approach is the incorporation of *in vitro* assays to test key events in the context of Adverse Outcome Pathways (AOPs), as it has been applied for skin sensitisation safety assessment (76). The currently limited database available for fish *in vitro* immunotoxicity assays would not support the identification of the most appropriate assays for such a tiered testing strategy. Also, AOPs that could integrate *in vitro* measurements of immune endpoints, comparable to the skin sensitisation AOP in humans, do currently not exist for fish. This does not mean that the application of *in vitro* testing for immunotoxicity assessment in fish is principally not possible, but it simply means that the existing database is too limited and it would need substantial and systematic research efforts to fill in the existing knowledge gaps. The recent years have seen substantial progress in the utilization of *in vitro* methodologies for ecotoxicological hazard assessment (280–282), but for the field of immunotoxicology we are not there yet.

## Author Contributions

HS wrote a first draft of the manuscript, which was then edited and further developed by KR, CB, and BJ. All authors have read and agreed to the published version of the manuscript.

## Funding

HS was supported by the grants 310030E-164266, 31003A_153427, and 31003A_130640 from the Swiss National Science Foundation SNSF. CB was funded by Swiss National Science Foundation (SNSF) Post Doc Mobility Fellowship number P400PB_183824. BJ was supported by the National Natural Science Foundation of China under the number 4197721.

## Conflict of Interest

The authors declare that the research was conducted in the absence of any commercial or financial relationships that could be construed as a potential conflict of interest.

## Publisher’s Note

All claims expressed in this article are solely those of the authors and do not necessarily represent those of their affiliated organizations, or those of the publisher, the editors and the reviewers. Any product that may be evaluated in this article, or claim that may be made by its manufacturer, is not guaranteed or endorsed by the publisher.
